# Theoretical Prediction Method for Tensile Properties of High-Strength Steel/Carbon Fiber-Reinforced Polymer Laminates

**DOI:** 10.3390/polym17070846

**Published:** 2025-03-21

**Authors:** Haichao Hu, Qiang Wei, Tianao Wang, Quanjin Ma, Shupeng Pan, Fengqi Li, Chuancai Wang, Jie Ding

**Affiliations:** 1 School of Mechanical Engineering, Tianjin Sino-German University of Applied Sciences, Tianjin 300350, China; huhaichao@tsguas.edu.cn (H.H.); 15631688218@163.com (T.W.); fengyinghezuo@foxmail.com (S.P.); fengqi18074837032@163.com (F.L.); wangchuancai@tsguas.edu.cn (C.W.); xuhain@126.com (J.D.); 2 School of Materials Science and Engineering, Hebei University of Technology, Tianjin 300401, China; 3 School of Mechanical Engineering, Hebei University of Technology, Tianjin 300401, China; 4 School of System Design and Intelligent Manufacturing, Southern University of Science and Technology, Shenzhen 518055, China

**Keywords:** composite laminate, Metal Volume Fraction theory, mechanical properties, numerical simulation, prediction

## Abstract

This study introduces a method for predicting the tensile properties of high-strength steel/carbon fiber-reinforced polymer (CFRP) composite laminates using Metal Volume Fraction (MVF) theory. DP590 and DP980 high-strength steels (thickness ~0.8 mm) were selected as substrates, and composite laminates were fabricated by compression molding with CFRP prepreg. Tensile tests were performed on an MTS universal testing machine, and fracture morphology was analyzed using scanning electron microscopy (SEM). The results demonstrated a typical mixed failure mode: necking and fracture in the metal layer, and neat fiber fracture in the CFRP layer. Comparisons of experimental tensile strength with theoretical predictions revealed that the model based on the metal strength at fracture significantly outperformed the model using tensile strength for predictions, with narrower error ranges. For example, the error for DP590/CFRP laminates ranged from 2.31% to 12.89%, whereas for DP980/CFRP laminates, it was –6.12%. Additionally, the study showed that using metals with higher plasticity in fiber metal laminates could underutilize the metal layer’s potential at peak stress, leading to significant deviations when predictions rely on tensile strength. Therefore, it is recommended to use the metal strength corresponding to peak stress for more accurate MVF-based tensile property predictions. This method provides a robust theoretical foundation for predicting the tensile performance of high-strength steel/CFRP laminates, aiding in optimizing structural designs for automotive and aerospace applications. Future research could explore the effects of different metal and fiber combinations, as well as more complex stacking designs.

## 1. Introduction

Since the 1990s, global energy supply constraints and growing uncertainties have prompted several countries and regions to initiate research programs related to material lightweighting [1,2]. However, as automotive lightweighting and passive safety requirements continue to evolve, relying solely on the development of metallic materials is no longer sufficient to meet the demanding standards of high-performance automotive design and manufacturing. Consequently, the development of advanced composite materials based on ultra-high-strength steels, aimed at further reducing structural weight, improving strength, and enhancing impact resistance and fatigue performance, has emerged as a research direction of significant engineering value [3,4]. Fiber metal laminates (FMLs), also referred to as Super Hybrid Laminates, are interlayer hybrid composite materials formed by alternating thin metal layers and fiber-reinforced layers, which are precisely cured to create a laminated structure [5]. FMLs combine the high strength of metals with the lightweight properties of fiber-reinforced composites, exhibiting excellent post-yield stiffness and stable “secondary stiffness” effects [6,7,8]. Compared to traditional metallic materials, FMLs offer lower density, superior fatigue and corrosion resistance, and enhanced flame retardancy [9]. In comparison with traditional fiber-reinforced composites, FMLs demonstrate better damage tolerance, notch strength, impact resistance, and adaptability to wet and humid environments. These unique properties make FMLs highly promising for applications in the aerospace and automotive industries [5,10,11].

Research on steel–fiber composite laminate structures both domestically and internationally has primarily focused on the aerospace and construction sectors. Studies on CFRP and metal laminates have mainly involved CARALL [12,13,14] and GLARE laminates [15,16,17], while in the construction sector, the application of fiber-reinforced steel materials has been the key area of research. Based on the MVF theory proposed by Vlot [18], which applies to unidirectional laminated FMLs, Ma Hongyi et al. [19] modified the formula to account for the characteristics of orthogonal laminates, enabling the prediction of the tensile properties of GLARE-3/2 laminates with 0°/0° and 0°/90° layups. Wang Yajie et al. [20] further examined the effect of fiber orientation on overall performance in orthogonal layups and, by combining the elastic modulus mixture rule for composites, modified the MVF theory to successfully predict the material’s elastic modulus, yield stress, and tensile strength. Moreover, the Metal Volume Fraction method based on the mixture rule can predict the tensile performance of FMLs with high accuracy. However, due to the significant elastoplastic behavior exhibited by the metal layer during stretching, simple elastic analysis cannot precisely capture its true tensile response.

Schmidt H. C. et al. [21] highlighted that combining local carbon fiber-reinforced plastic (CFRP) and metal sheets can form highly efficient lightweight structures, which are expected to significantly reduce weight and greenhouse gas emissions. Meanwhile, studies by Guo Y. and Zhai C. [22] indicate that U-shaped and box-shaped structures made from steel/CFRP composites possess higher load-bearing capacity and superior energy absorption characteristics compared to single-material structures. Layered composite materials, with their lightweight and high-strength properties, are widely used in deformable components such as floor panels, engine hoods, roofs, and door panels in the automotive industry, as well as in structural applications like tail fins, wings, and fuselages in aerospace. Among these, steel/polymer/steel sandwich composites stand out due to their high rigidity, high strength, excellent formability, and good vibration-damping properties, offering significant advantages in structural design. Additionally, special steel covers demonstrate excellent corrosion resistance [23]. Luca Quagliato et al. [24,25], through their study of the manufacturing process for thin steel-plate-carbon fiber-reinforced polymer core sandwich laminates, quantitatively analyzed their mechanical properties and fracture behavior, finding that the bending performance of this composite material was improved by 48% compared to pure steel materials.

In summary, high-strength steel–carbon fiber composite structures, with their outstanding mechanical properties, demonstrate broad application prospects in the automotive and rail transportation industries, among others. In this study, high-strength steel–carbon fiber composite laminates were designed and fabricated based on the compression molding process. The microscopic structural characteristics of these laminates were tested and analyzed, and their tensile properties were predicted based on the MVF theory. Furthermore, this study investigated the effect of different steel plate thicknesses on the tensile strength of the laminates and conducted a systematic analysis in conjunction with existing tensile performance prediction models for high-strength steel/CFRP composite laminates.

This study introduces two key advancements in MVF theory:

(1) A dynamic metal strength criterion based on real-time laminate fracture stress, addressing strain incompatibility limitations in conventional models;
(2) A quantitative error analysis linking prediction accuracy to metal-to-fiber ratios.

These innovations aim to improve the design of hybrid laminates with high plasticity metals.

## 2. Materials and Methods

### 2.1. Material Preparation for Experiments

The study selected two high-strength steel materials, DP590 and DP980, each approximately 0.8 mm in thickness, as the metal substrates. These DP series materials are dual-phase steels, and the combining of soft and hard phases in these materials provides good forming properties and strength. The steel plate dimensions used in the experiments were typical dimensions: 480 mm × 450 mm. The chemical composition of these materials is provided in Table 1. Additionally, the steel materials used in this study were sourced from Beijing Shougang Group, located in Beijing, China.

Table 2 provides the yield strength, tensile strength, and elongation of DP590 and DP980 under room temperature conditions.

The type of prepreg used in the experiment was C8142U0-125, with the specific parameters detailed in Table 3, Table 4 and Table 5.

The CFRP prepreg (C8142U0-125) comprised Toray T700S carbon fibers with 60% fiber volume fraction in an epoxy L20 matrix (elastic modulus = 3.2 GPa, Poisson’s ratio = 0.35). The fiber–matrix interface was enhanced using 0.5 wt% silane coupling agent (γ-glycidoxypropyltrimethoxysilane).

### 2.2. Test Plate Preparation

The preparation process of the test plates involves the following steps: First, the prepreg is conditioned for approximately 0.5 h to ensure the complete resin impregnation of the fibers, achieving optimal molding results. Next, the prepreg and steel plates are cut according to the design requirements and arranged according to the stacking design. The materials are then placed in a mold, where heating and pressurizing are applied to cure and form the composite. Finally, after the mold cools to room temperature, the test plate is removed, completing the preparation.

**Figure 1:**
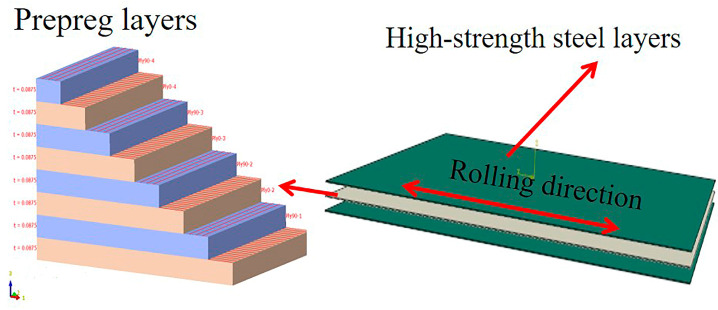
The schematic diagram of the high-strength steel/CFRP composite laminate structure.

Figure 1 presents a schematic diagram of the high-strength steel/CFRP composite laminate structure, illustrating the layering and composition of the composite material.In the stacking design, a simple symmetric stacking method is used to ensure the mechanical properties and stability of the composite material, for example, [0°/90°]*_n_*, where 0° and 90° represent the fiber orientation angle with the reference coordinate axis, and n represents the number of repetitions of the stacking combination. Specifically, [0°/90°]*_n_* refers to a laminate composed of n layers of unidirectional carbon fibers alternately stacked at 0° and 90° orientations. For example, [0°/90°]*_4_* refers to a laminate made up of 4 layers of unidirectional carbon fibers alternately stacked at 0° and 90° orientations. This stacking method is typically used in structures subjected to biaxial loading to improve the mechanical performance of the composite in different directions.

**Figure 2:**
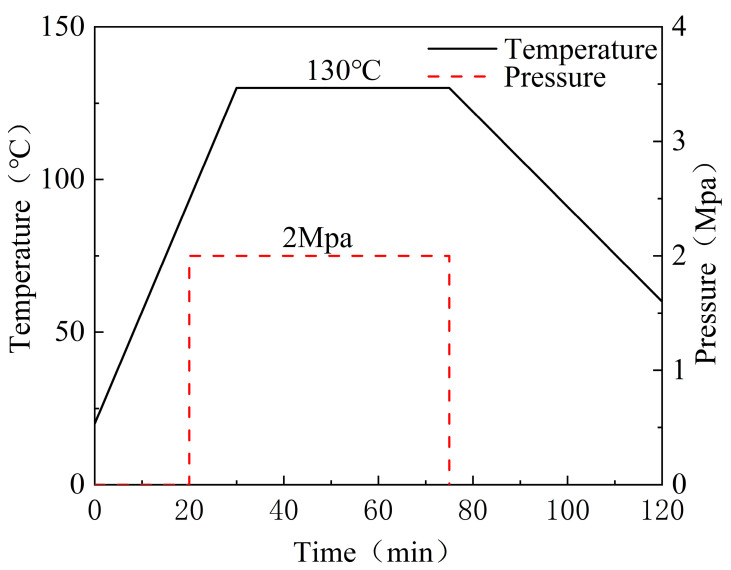
Hot press curing process.

**Figure 3:**
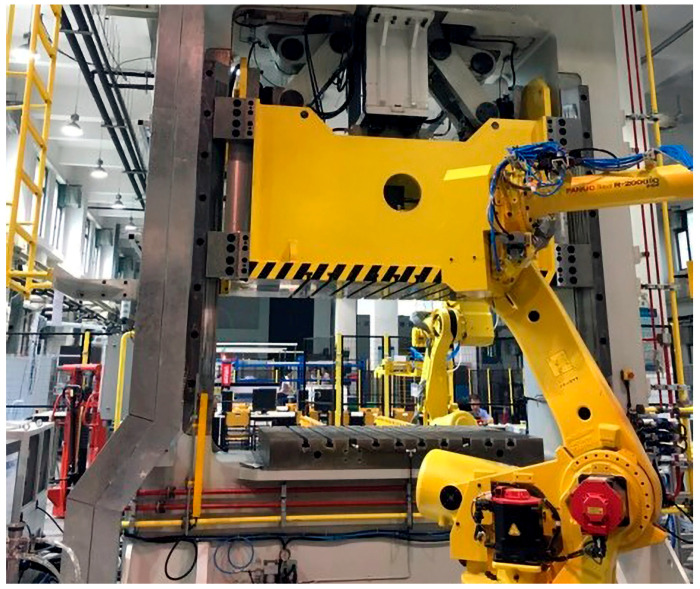
Mold pressure-forming equipment.

The compression molding process was conducted at 130 °C under 2 MPa pressure for 60 min, followed by a controlled cooling rate of 2 °C/min to minimize residual stresses. Post-fabrication quality checks via ultrasonic C-scan revealed <1% void content across all laminates. The compression molding process is shown in Figure 2, and the mold pressure-forming equipment is shown in Figure 3.

### 2.3. Performance Testing

**Figure 4:**
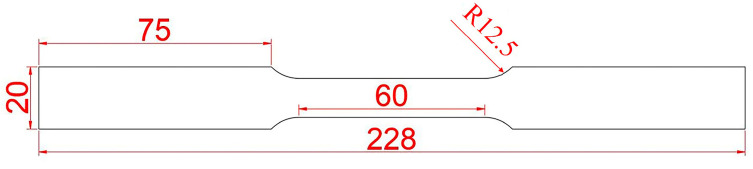
Schematic diagram of the specimen dimensions.

According to the ASTM D638 standard [30], the prepared high-strength steel/carbon fiber-reinforced polymer (CFRP) laminates were processed into standard specimens, as shown in Figure 4. Tensile tests were conducted on an MTS universal testing machine, sourced from MTS Systems Corporation, Eden Prairie, Minnesota, USA. After the tensile tests, the fracture surfaces of the specimens were sectioned, and the carbon fiber layers were gold-coated. The fracture morphology was then observed using a SUPRA™ 55 scanning electron microscope, manufactured by Carl Zeiss AG, Oberkochen, Germany.

### 2.4. Introduction to MVF Fundamental Theory

Fiber metal laminates (FMLs) exhibit significant anisotropic characteristics in their mechanical properties, which are closely related to the intrinsic properties of fiber-reinforced composites. For the prediction of the mechanical properties of fiber–aluminum alloy composite structures, researchers such as Vlot et al. established a quantitative analysis model based on the Metal Volume Fraction (MVF) theory. Studies indicate that when the MVF value of the material system falls within the range of 0.45–0.85, the theoretical predictions show good agreement with experimental data. The MVF defined by Vlot et al. is expressed by Equation (1) as follows:

(1) $$MVF=\sum_{i}^{n}\frac{t_{al}}{t_{lam}}$$

In the equation, $t_{lam}$ represents the thickness of the fiber metal laminate; $t_{al}$ denotes the thickness of a single aluminum alloy layer; and n is the number of aluminum alloy layers. Building upon this definition, Vlot et al. further proposed a predictive formula for the tensile properties of GLARE:

(2) $$E_{lam}=MVF\times E_{met}+\left(1-MVF\right)\times E_{FRP}$$

(3) $$\sigma_{t,lam}=MVF\times \sigma_{t,met}+(1-MVF)\times \sigma_{t,FRP}$$

In the equation, $\sigma_{t}$ represents the tensile ultimate strength; E represents the tensile modulus; the subscript lam denotes the laminate; the subscript met denotes the metal layer; and the subscript FRP denotes the fiber-reinforced composite material.

According to the MVF theory, the strength of the composite laminate involved in this paper can be calculated using the following equation:

(4) $$\sigma_{lam}=\frac{\sigma_{Metal}*\sum_{1}^{P}t_{metal}+\sigma_{CFRP\left(90^{{}^{\circ}}\right)}*\sum_{1}^{P}t_{CFRP\left(90^{{}^{\circ}}\right)}+\sigma_{CFRP\left(0^{{}^{\circ}}\right)}*\sum_{1}^{p}t_{CFRP\left(0^{{}^{\circ}}\right)}}{t_{total}}$$

In this equation,

$\sigma_{lam}$—Theoretical value of tensile strength of laminates;

$\sigma_{Metal}$—Tensile strength value of the metal layer;

$\sigma_{CFRP(90{}^{\circ})}$—CFRP layer 90° strength value;

$\sigma_{CFRP(0{}^{\circ})}$—CFRP layer 0° strength value.

Our prediction methodology is based on the Metal Volume Fraction (MVF) theory, which estimates the mechanical properties of composite materials by considering the volume fractions and mechanical properties of their constituent phases. The steps are as follows:

CFRP Layer Characterization:

Strength Acquisition: The longitudinal and transverse strengths of the CFRP layer are obtained from Table 5.

**Figure 5:**
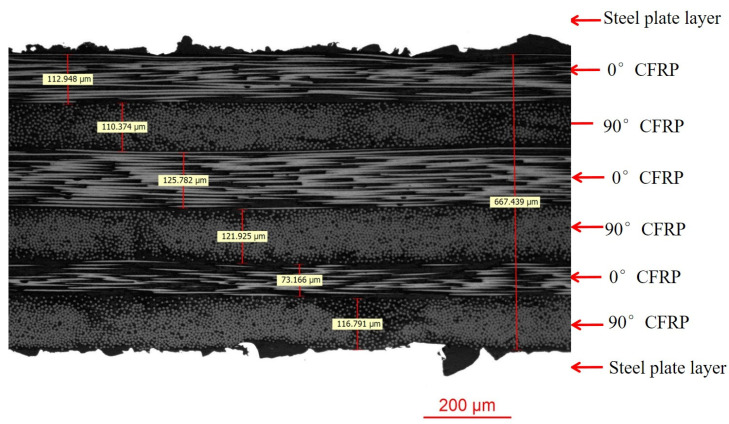
Typical metallographic interface of high-strength steel/carbon fiber composite laminate.

Thickness Measurement: The average thickness of the CFRP layer is determined from Figure 5.

Metal Layer Characterization:

Thickness Definition: Each metal layer has a thickness of 0.8 mm.

Strength Acquisition: The yield and tensile strengths of the high-strength steel are retrieved from Table 2. For the specific case of CFRP laminate fracture, the corresponding strength of the high-strength steel is obtained fromthe tensile stress–strain curve of High-Strength Steel/CFRP composite laminate specimens. 

Model Implementation and Strength Prediction:

MVF Model Application: The MVF model is applied, utilizing the acquired CFRP and metal layer properties to predict the overall strength of the composite structure. The model calculates the contribution of each phase to the total strength based on their respective volume fractions and mechanical properties.

Validation: The predictions are validated against experimental data to ensure accuracy and identify any discrepancies for further investigation.

### 2.5. Different High-Strength Steel/CFRP Composite Laminate Structural Designs

To investigate the influence of high-strength steel types and the number of carbon fiber layers on the mechanical properties of high-strength steel/CFRP composite laminates, this study designed six groups of such structures. The specific structural designs of these groups are presented in Table 6, which provides an overview of the different design options for these advanced composite structures.

## 3. Results and Discussion

### 3.1. High-Strength Steel/Carbon Fiber Composite Laminate Interface Structure

The interface between high-strength steel and carbon fiber-reinforced polymer (CFRP) is a critical factor influencing the overall mechanical properties of composite laminates. A detailed microstructural analysis is required to understand the formation of the interface microstructure and its impact on material performance.

Figure 5 presents the structural characteristics of the high-strength steel/carbon fiber composite laminate interface under metallographic observation. In this figure, the carbon fiber layers consist of six layers with an overall thickness of 667.439 μm. The thickness of each layer is relatively similar, with a minimum thickness of 73.166 μm and a maximum thickness of 125.782 μm. The thickness distribution shows some fluctuation, with an average thickness of 112.23 μm. In subsequent strength predictions, the average thickness of the fiber layers is used as the reference.

**Figure 6:**
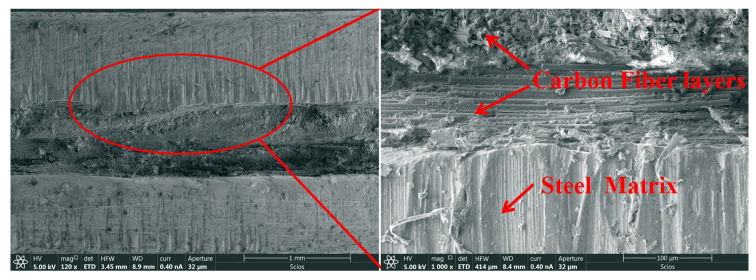
Typical SEM interface of high-strength steel/carbon fiber composite laminate.

Figure 6 reveals the structural characteristics of the high-strength steel/carbon fiber composite laminate interface under scanning electron microscopy (SEM) observation. The figure reflects the bonding features of the microscopic interface between the fiber layers and the high-strength steel. From this figure, it can be observed that the 0° carbon fiber layers, 90° carbon fiber layers, resin matrix, and metal matrix are densely bonded. The interface between the metal matrix and the carbon fiber layers is filled with resin, providing effective adhesion. The microstructure shows no significant forming defects, indicating that the molding process is suitable for the preparation of high-strength steel–carbon fiber composite laminates.

**Figure 7:**
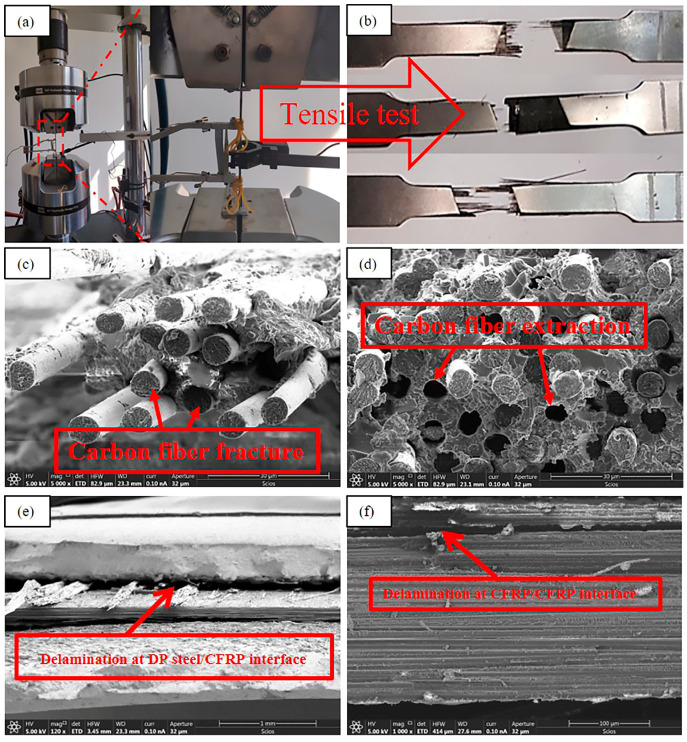
SEM image of the fiber layer fracture in high-strength steel/CFRP composite laminate (**a**) Schematic Diagram of Tensile Testing Device; (**b**) Typical Tensile Failure Specimen; (**c**) Typical Carbon Fiber Fracture Cross-Section; (**d**) Typical Fiber Pull-Out Cross-Section; (**e**) Delamination Between DP Steel and CFRP Layers; (**f**) Interlaminar Delamination in CFRP lays.

Figure 7 presents comprehensive SEM images illustrating the complex failure mechanisms in the high-strength steel/CFRP composite laminate. Figure 7a,b provide an overview of the tensile failure, with the metal layer exhibiting pronounced necking and fracture. Figure 7c,d focus on the carbon fiber layer, revealing typical failure modes such as clean fiber breakage and fiber pull-out. Figure 7e,f emphasize interfacial and intra-layer failures, highlighting debonding between the CFRP layer and the steel plate, as well as delamination within the CFRP layer itself. Collectively, these images illustrate a mixed-mode failure mechanism in the composite laminate, characterized by metal necking and fracture, fiber breakage and pull-out, interfacial debonding, and intra-layer delamination.

### 3.2. Comparison and Analysis of Stress–Strain Curves Between High-Strength Steel/CFRP Composite Laminates and High-Strength Steel

**Figure 8:**
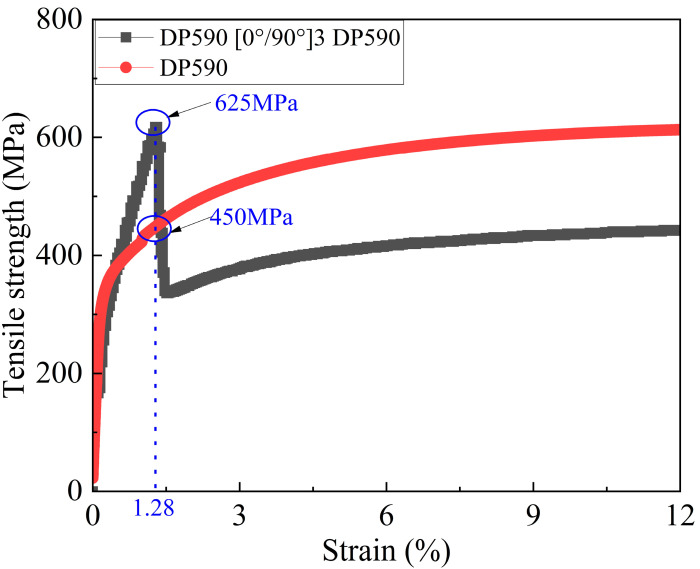
Tensile stress–strain curve of DP590/CFRP composite laminate specimen.

As shown in Figure 8, during the loading of the high-strength steel/CFRP composite laminate tensile specimen, the stress rapidly increases to its peak value as the strain gradually increases. When the strain reaches 1.28%, the stress quickly rises to the peak value. At this point, the stress in the high-strength steel layer is approximately 450 MPa, which is far below its ultimate stress, indicating that the carbon fiber layer has reached its limit and is about to fracture. Following this, after the composite laminate reaches its peak stress, the stress quickly drops to 325 MPa due to the fracture of the carbon fiber layer. Subsequently, as the strain further increases, the stress of the specimen gradually rises, with the primary load-bearing responsibility being taken over by the DP590 metal layer. Furthermore, from the tensile stress–strain curve of the DP590/CFRP composite laminate, it can be observed that the strain value corresponding to its tensile strength is significantly shifted forward compared to the strain value corresponding to the tensile strength of the DP590 monolithic material, indicating that the overall structure exhibits a certain secondary stiffness characteristic.

**Figure 9:**
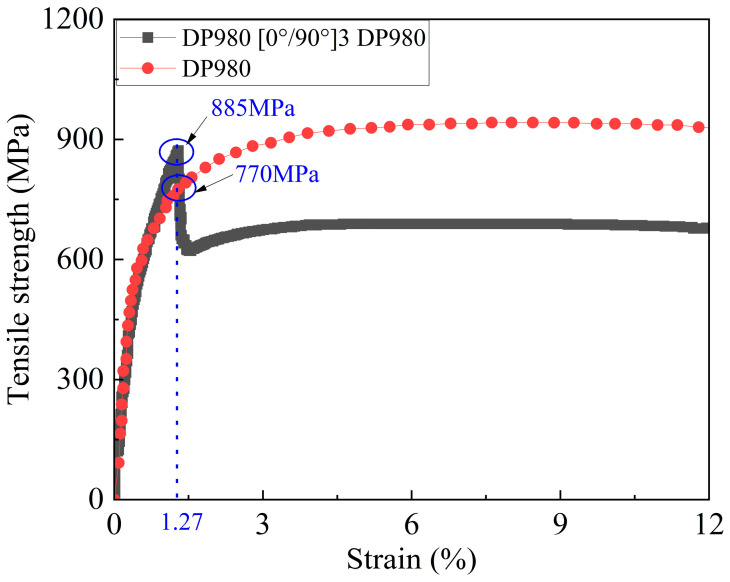
Tensile stress–strain curve of DP980/CFRP composite laminate specimen.

As shown in Figure 9, during the loading process of the DP980/CFRP composite laminate tensile specimen, the stress rapidly increases to its peak value as the strain gradually increases. When the strain reaches 1.27%, the stress quickly rises to the peak value, and the strength of the composite laminate is approximately 885 MPa. Given that this strain level is far below the strain required for the fracture of high-strength steel, and the distinct sound of fiber layer fracture is clearly audible during the experiment, it can be inferred that the carbon fiber layer has reached its ultimate stress and has fractured at this point.

After the fiber layer fractures, the overall stress of the composite laminate rapidly drops to 770 MPa. Subsequently, as the strain of the composite laminate continues to increase, the specimen’s stress gradually rises, with the stress increase mainly being provided by the metal layer. Meanwhile, the tensile stress–strain curve of the DP980/CFRP composite material shows that the strain value corresponding to its tensile strength is significantly shifted forward compared to that of the DP980 monolithic material.

### 3.3. Prediction of Tensile Properties of Composite Laminate

This section explores the application of the Metal Volume Fraction (MVF) theory to predict the overall strength of high-strength steel/carbon fiber-reinforced polymer (CFRP) laminate composites. Given that DP-series high-strength steel exhibit significant plasticity and that the mechanical properties of the composite material are influenced by the interaction between the metal and fiber layers, we will focus on the yield strength and tensile strength of high-strength steel, as well as the impact of these strengths on the overall strength prediction of the composite laminate at failure. The interaction between high-strength steel and CFRP plays a critical role in the mechanical behavior of the laminate, and accurately describing this interaction is essential in improving the reliability of composite material design.

Table 7 shows the comparison between the predicted strength values based on the yield strength of high-strength steel and the experimental values. The error analysis reveals that the minimum error is 1.0562 MPa (sample 4) and the maximum error is 116.5625 MPa (sample 6), with the error range spanning from 1.06 to 116.56 MPa. In general, the predicted values for samples 1–4 are lower than the experimental values, with an error range from −9.24% to −0.15%. This indicates a tendency to underestimate in the theoretical predictions based on yield strength in this range. However, for samples 5 and 6, where the number of carbon fiber layers is higher, the predictions are overestimated, with sample 5 showing an error of 6.60% and sample 6 showing an error of −13.08%. Overall, the theoretical predictions based on yield strength tend to be conservative when the number of carbon fiber layers is low but may overestimate the strength of the composite laminate when the number of carbon fiber layers is high, suggesting the need for the further optimization of the prediction model to improve its applicability.

Table 8 shows the comparison between the predicted strength values based on the tensile strength of high-strength steel and the experimental values. The error analysis indicates that the minimum error is 48.4375 MPa (sample 6) and the maximum error is 167.657 MPa (sample 1), with the error range between 48.44 MPa and 167.66 MPa. From the overall trend, it is clear that all the predicted values are significantly higher than the experimental values, with an error range of 5.44% to 33.93%. This indicates a systematic overestimation issue with the prediction method based on tensile strength. Particularly for samples 1–5, the errors exceed 20%, with sample 1 showing the highest error at 33.93%. The systematic overestimation (20–34%) in Table 8 arises because the tensile strength of metals (e.g., DP590: 592 MPa) assumes full plasticity development, whereas the CFRP layer fractures at 1.28% strain (Figure 8), limiting metal stress to ~450 MPa (76% of its tensile strength). This discrepancy highlights a fundamental limitation of traditional MVF models that neglect strain incompatibility between phases. Future models should incorporate strain-dependent metal strength degradation to improve accuracy. Although the error for sample 6 is relatively small (5.44%), it still shows some degree of overestimation. Comprehensive analysis suggests that the prediction method based on tensile strength may not fully account for the structural characteristics of the composite laminate and the actual stress distribution, leading to a systematic overestimation of the theoretical predictions. The model needs further adjustment to improve the prediction accuracy.

Table 9 shows the comparison between the predicted composite laminate strength values based on the strength of high-strength steel at failure and the experimental values. The error analysis reveals that the minimum error is 11.407 MPa (sample 1), and the maximum error is 88.674 MPa (sample 5), with the error range between 11.41 MPa and 88.67 MPa. Overall, the theoretical prediction values for samples 1–5 are higher than the experimental values, with an error range from 2.31% to 12.89%. As the number of carbon fiber layers increases, the error gradually increases, indicating that the prediction method exhibits smaller errors for laminates with fewer carbon fiber layers but a tendency to overestimate when the number of carbon fiber layers is higher. Sample 6, however, shows a predicted value lower than the experimental value with an error of −6.12%, suggesting that the method may have some deviation in its applicability to high-strength materials like DP980. The novelty of selecting metal strength at fracture lies in its ability to capture the dynamic load transfer between phases. As shown in Figure 8, the CFRP fracture occurs when the steel layer is only at 450 MPa (75% of DP590’s tensile strength), demonstrating that traditional MVF models overestimate by assuming full metal utilization (e.g., 33.93% error in Table 8). In summary, the method based on the strength of high-strength steel at laminate failure shows smaller errors for lower-layer composites but may overestimate the strength for composites with more carbon fiber layers and may underestimate the strength when the metal substrate has higher strength. Therefore, further optimization of the prediction model is needed to improve its applicability for different layer numbers and materials.

The observed prediction errors (e.g., 12.89% for DP590 [0°/90°]*_5_* DP590) primarily stem from residual thermal stresses induced during post-molding cooling. According to classical laminate theory, the DP590/CFRP laminates develop compressive residual stresses of approximately 85 MPa in the steel layer due to mismatched thermal expansion coefficients (CFRP: 2 × 10^−6^/°C vs. steel: 12 × 10^−6^/°C). These stresses counteract the applied tensile load, effectively reducing the laminate’s load-bearing capacity and introducing systematic overestimation in theoretical predictions. From the above analysis, it can be observed that when preparing metal–fiber composite laminates using metals with high plasticity, it is crucial to consider the phenomenon where the potential of the metal layer has not been fully released when the laminate reaches its stress peak. If the tensile strength of the metal is directly used as a parameter and applied in the MVF theory for predictions, significant errors may arise. Therefore, it is recommended that when applying the MVF theory to predict the tensile properties of composite laminates, the metal strength parameter corresponding to the laminate at the stress peak should be selected in order to improve the accuracy and reliability of the predictions.

### 3.4. Discussion on Failure Mechanism

The failure mechanisms of DP steel/CFRP laminates exhibit distinct dependencies on metal plasticity and fiber reinforcement ratios. Building on the experimental results in Figure 7, Figure 8 and Figure 9 and Table 9, we analyze the failure hierarchy through two critical regimes:

DP590/CFRP: Dominant fiber-to-metal interface debonding occurs due to moderate stiffness mismatch (DP590: E = 210 GPa; CFRP: E1 = 125 GPa). The high plasticity of DP590 allows partial stress redistribution, but CFRP fracture at <1.3% strain (Figure 8) limits metal stress to approximately 76% of its tensile strength (Table 9).

(1) Low Fiber Ratio (1–3 CFRP Layers)

DP980/CFRP: Higher yield strength (666 MPa 666 MPa) induces localized plastic constraints, promoting interfacial delamination (Figure 7b).

(2) High Fiber Ratio (4–5 CFRP Layers)

Increased CFRP stiffness dominates load transfer, suppressing metal plasticity. Residual thermal stresses (∼85 MPa∼85 MPa, Section 3.3) exacerbate strain localization, leading to brittle CFRP fractures with minimal metal contribution (error increases to 12.89%12.89%, Table 9).

(3) Mechanism Summary

Metals with higher plasticity (e.g., DP590, 28.5% 28.5% elongation) exhibit greater stress underutilization due to CFRP’s early failure.

Lower plasticity metals (e.g., DP980) prioritize interface-driven failure but achieve better stress utilization (−6.12%−6.12% error, Table 9).

## 4. Conclusions

This study established an optimized framework for predicting the tensile properties of steel/CFRP laminates through MVF theory refinement and experimental validation. The principal findings are summarized as follows:

### 4.1. MVF Model Optimization

Utilizing metal strength at fracture significantly improves the prediction accuracy, reducing errors to 2.31–12.89% for DP590/CFRP laminates and −6.12% for DP980/CFRP laminates (Table 9). In contrast, traditional models based on tensile strength exhibit substantially larger errors (5.44–33.93%, Table 8), demonstrating the critical importance of accounting for incomplete metal plasticity utilization.

### 4.2. Failure Mode Transition

CFRP-dominated failure occurs at <1.3% strain (Figure 8 and Figure 9), far below the fracture strain of monolithic steel (e.g., DP590: 28.5%, Table 2). This early CFRP fracture limits the metal layer’s ability to reach its full tensile strength, highlighting the necessity of strain compatibility analysis in hybrid laminate design.

### 4.3. Process–Structure Linkage

The compression molding process with controlled cooling (2 °C/min) minimizes residual stresses to <85 MPa, as validated by interfacial SEM analysis (Figure 6). This process stability ensures consistent bonding quality, which is pivotal for aligning experimental results with theoretical predictions.

These findings provide a scientifically robust foundation for designing high-performance steel/CFRP laminates in automotive and aerospace applications, while emphasizing the need for strain-dependent MVF model calibration in future studies.

## Figures and Tables

**Table 1:** Main chemical compositions (%, by mass) of DP590 and DP980.

	C	Mn	Si	P	S	Al	Cr	Fe
DP590	0.11	1.81	0.58	0.011	0.003	-	-	Bal
DP980	0.19	2.07	0.13	0.01	0.001	0.05	0.1	Bal

**Table 2:** The performance parameters of DP590 and DP980.

	Tensile Strength (MPa)	Yield Strength (MPa)	Rate of Elongation/%
DP590	592	340	28.5
DP980	996	666	12

**Table 3:** The characteristic parameters of the prepreg resin matrix.

Test Item	Performance Index
Chemical property	Thermosetting epoxy resin
Curing temperature	130~150 °C
Gelation time	18~20 min@130 °C
	9~11 min@140 °C
	5~7 min@150 °C
Glass transition temperature (Tg)	95 °C

**Table 4:** Characteristic parameters of prepreg.

Test Item	Performance Index
Form of establishment	UD
Fiber surface density(g/m^2^)	125 ± 6
Prepreg surface density(g/m^2^)	215 ± 8
Resin content	42 ± 2

**Table 5:** The prepreg corresponds to the characteristic parameters of the laminate.

Test Item	Performance Index	Test Standard
0° Tensile strength	1800 (Mpa)	ASTM D3039 [26]
90° Tensile strength	50 (Mpa)	ASTM D3039 [26]
Bending strength	1100 (Mpa)	ASTM D7264 [27]
Bending modulus	110 (Gpa)	ASTM D7264 [27]
Interlaminar shear strength	65 (Mpa)	ASTM D2344 [28]
Longitudinal modulus (E1)	125 (Gpa)	ASTM D3039 [26]
Transverse modulus (E2)	8.5 (Gpa)	ASTM D3039 [26]
Shear modulus (G12)	4.2 (Gpa)	ASTM D3518 [29]

**Table 6:** Different high-strength steel/CFRP composite laminate structural designs.

No.	Structures
1	DP590 [0°/90°]*_1_* DP590
2	DP590 [0°/90°]*_2_* DP590
3	DP590 [0°/90°]*_3_* DP590
4	DP590 [0°/90°]*_4_* DP590
5	DP590 [0°/90°]*_5_* DP590
6	DP980[0°/90°]*_3_* DP980

**Table 7:** Comparison of the predicted strength values and experimental values of the composite laminate based on the yield strength of high-strength steel.

NO.	Structures	Experimental Tensile Strength (MPa)	Yield Theory Predicts Strength (MPa)	Error Rate
1	DP590 [0°/90°]*_1_* DP590	494.093	448.4167	−9.24%
2	DP590 [0°/90°]*_2_* DP590	583.934	550.1429	−5.79%
3	DP590 [0°/90°]*_3_* DP590	645.7135	626.4375	−2.99%
4	DP590 [0°/90°]*_4_* DP590	686.834	685.7778	−0.15%
5	DP590 [0°/90°]*_5_* DP590	687.826	733.25	6.60%
6	DP980[0°/90°]*_3_* DP980	891	774.4375	−13.08%

**Table 8:** Comparison of the predicted strength values and experimental values of the composite laminate based on the tensile strength of high-strength steel.

NO.	Structures	Experimental Tensile Strength (MPa)	Tensile Strength Prediction Value (MPa)	Error Rate
1	DP590 [0°/90°]*_1_* DP590	494.093	661.75	33.93%
2	DP590 [0°/90°]*_2_* DP590	583.934	733	25.53%
3	DP590 [0°/90°]*_3_* DP590	645.7135	786.4375	21.79%
4	DP590 [0°/90°]*_4_* DP590	686.834	828	20.55%
5	DP590 [0°/90°]*_5_* DP590	687.826	861.25	25.21%
6	DP980[0°/90°]*_3_* DP980	891	939.4375	5.44%

**Table 9:** Comparison of the predicted composite laminate strength values and experimental values based on the strength of high-strength steel at laminate fracture.

NO.	Structures	Experimental Tensile Strength (MPa)	Fracture Theoretical Predicted Strength (MPa)	Error Rate
1	DP590 [0°/90°]*_1_* DP590	494.093	505.5	2.31%
2	DP590 [0°/90°]*_2_* DP590	583.934	602.29	3.14%
3	DP590 [0°/90°]*_3_* DP590	645.7135	674.875	4.52%
4	DP590 [0°/90°]*_4_* DP590	686.834	731.33	6.48%
5	DP590 [0°/90°]*_5_* DP590	687.826	776.5	12.89%
6	DP980[0°/90°]*_3_* DP980	891	836.4375	−6.12%

## Data Availability

The original contributions presented in this study are included in the article material. Further inquiries can be directed to the corresponding authors.
